# Characterization of the effect of MRI on Gafchromic film dosimetry

**DOI:** 10.1120/jacmp.v16i6.5743

**Published:** 2015-11-08

**Authors:** Meral L. Reyhan, Ting Chen, Miao Zhang

**Affiliations:** ^1^ Department of Radiation Oncology Rutgers Cancer Institute of New Jersey New Brunswick NJ USA

**Keywords:** Gafchromic film, MRI, MRI linac, ViewRay

## Abstract

Magnetic resonance (MR) imaging of Gafchromic film causes perturbation to absolute dosimetry measurements; the purpose of this work was to characterize the perturbation and develop a correction method for it. Three sets of Gafchromic EBT2 film were compared: radiation (control), radiation followed by MR imaging (RAD+B), and MR imaging followed by radiation (B+RAD). The T1‐weighted and T2‐weighted MR imaging was performed using a 1.5T scanner with the films wedged between two chicken legs. Doses from 0 to 800 cGy were delivered with a 6MV linac. The time interval between radiation and MR imaging was less than 10 min. Film calibration was generated from the red channel. Microscopic imaging was performed on two pieces of film. The effect of specific absorption rate (SAR) was determined by exposing another three sets of films to low, medium, and high levels of SAR through a series of pulse sequences. No discernible preferential alignment was detected on the microscopic images of the irradiated film exposed to MRI. No imaging artifacts were introduced by Gafchromic film on any MR images. On average, 4% dose difference was observed between B+RAD or RAD+B and the control, using the same calibration curve. The pixel values between the B+RAD or RAD+B and the control films were found to follow a linear relationship pixel(Control)=1.02×pixel(B+RAD or RAD+B). By applying this correction, the average dose error was reduced to approximately 2%. The SAR experiment revealed a dose overestimation with increasing SAR even when the correction was applied. It was concluded that MR imaging introduces perturbation on Gafchromic film dose measurements by 4% on average, compared to calibrating the film without the presence of MRI. This perturbation can be corrected by applying a linear correction to the pixel values. Additionally, Gafchromic film did not introduce any imaging artifacts in any of the MR images acquired.

PACS number: 87.50.cm

## I. INTRODUCTION

Gafchromic film (Ashland Inc., Covington, KY) has been widely used in radiation oncology for dosimetric quality assurance (QA) testing, 2D dose verification, and in‐vivo dosimetry.^1^, ^2^, ^3^, ^4^ It is a self‐developing film with high spatial resolution and low energy dependency. Gafchromic film (EBT2) is composed of multiple layers: polyester laminate, adhesive, active, and polyester. The active layer is composed of lithium pentacosa‐10,12‐diynoate, LiPCDA. The rod‐shaped crystals of the active layer preferentially align in a down‐web direction during the coating process, which increases the sensitivity of the dosimeter.^5^, ^6^

When LiPCDA is exposed to ionizing radiation polymerization occurs. The polymerization has two phases. The fast polymerization phase starts within 100 microseconds of exposure and proceeds for about 30 milliseconds. Within a second the initial fast phase converts to a slow phase.^(5)^ The slow phase leads to post irradiation development, which stabilizes after 2 hr, though a 24‐hr time frame is recommended to mitigate this effect. Using proper film handling and storage techniques, accurate reference dosimetry measurements can be made with an uncertainty of ±2% using EBT2 film.^(7)^

The University Medical Center Utrecht, in cooperation with Elekta (Elekta, Stockholm, Sweden) and Philips (Philips Healthcare, Best, The Netherlands), has developed a combined 1.5T magnetic resonance imaging (MRI)‐linear accelerator system, enabling soft‐tissue‐based image guidance and treatment response monitoring.^8^, ^9^ ViewRay (ViewRay Inc., Cleveland, OH) uses a combination of 0.35‐Tesla magnetic resonance imaging (MRI) and cobalt‐60 (^60^Co) radiotherapy to enable soft‐tissue‐based image guidance and treatment plan adaptivity.^(10)^ Both modalities make use of high magnetic fields in conjunction with radiation therapy machines. Radiation dosimeters capable of absolute dosimetry measurements under strong magnetic and radiofrequency fields would be desirable for quality assurance and *in vivo* dosimetry measurements for use with these modalities. *In vivo* measurements would necessitate accurate dose measurements without inducing MR imaging artifacts.

Gafchromic film has previously been used for relative dosimetry measurements of beam profiles under high magnetic field conditions. However, 1%–4% lower dose levels were observed when comparing absolute dosimetric measurements made in 0T to 0.6 and 1.3T magnetic fields.^(8)^ In this paper, characterization of the dosimetric difference denoted between measurements made with and without magnetic fields and a method of correction for the presence of the magnetic field are presented.

## II. MATERIALS AND METHODS

### A. Experimental design

Three sets of 24 pieces of EBT2 Gafchromic film were irradiated. The first set was used as a control and not exposed to a magnetic field. The second set of film (RAD+B) was irradiated prior to entering the magnetic field of the MRI system. The third set of film (B+RAD) was exposed to the magnetic field of the MRI system, and then irradiated. Irradiation took approximately 30 min to complete, starting with the highest dose. The time gap between irradiation and exposure to MRI was minimized to 10 min. By exposing the film to MRI both before and after irradiation we hoped to mimic a clinical MRI‐radiotherapy configuration. It was hypothesized that by placing the films in the high magnetic field changes to the alignment of the active layer might occur, which would ultimately affect the polymerization process. By placing the film in the magnetic field before irradiation, the changes to the fast polymerization process might be observed, whereas by placing the film in the magnetic field after irradiation, while the slow polymerization process was underway, characterization of polymerization while in a magnetic field could be studied.

### B. Film calibration

Gafchromic EBT2 film (lot A091710003) was cut into 1 cm by 1.25 cm strips. A calibrated 6 MV photon beam from a 21EX Clinac (Varian Medical Systems, Palo Alto, CA) was used to deliver 0, 5, 10, 25, 50, 100, 150, 200, 250, 300, 500, and 800 cGy to two pieces of the cut film. All films were scanned by an Epson Expression 1000XL flatbed scanner (Epson, Suwa, Japan) 24 hr after irradiation. The transmissive scan setting was used in conjunction with settings for 48‐bit color and a 300 dpi resolution. Mean pixel value from the red channel (RC) was determined using an in‐house software^(11)^ from Tiff images. A polynomial fit was used to convert the mean pixel value from the two irradiated film pieces to dose. No off‐axis lateral corrections were used as films were placed within 3.2 cm of the center of the Epson scanner.

### C. Microscopic imaging

Microscopic imaging was performed on two pieces of film to determine if preferential alignment of the polymers occurred after exposure to the magnetic field. One film was exposed to the magnetic field of the MRI for 20 min and irradiated within 5 min of removal from the MRI machine to a dose of ∼500 cGy. The other film received equivalent dose, but was not exposed to the magnetic field. The top polyester layer was partially removed from both pieces of film for microscopic imaging. Olympus BX41 transmitted‐light microscope (Olympus Corp., Tokyo, Japan) coupled with Olympus MicroFire CCD camera system was used to acquire the image, with a magnification of 40X.

### D. Magnetic resonance imaging

A 1.5T GE Signa HDxt MRI (GE Healthcare, Waukesha, WI) was used to image the Gafchromic film. The film was placed between two pieces of chicken to simulate an *in vivo* dosimetry setup. The GE 8‐channel cardiac coil (GE Healthcare, Little Chalfont, UK) was used for imaging and a three‐plane localizer FIESTA sequence was used to determine the location of the film with the following sequence parameters: 50° flip angle, 8 mm slice thickness, repetition time of 3.233 ms, echo time of 1.352 ms, bandwidth of 976.6 Hz/px, and $1.5625\times 1.5625\;{\text{mm}}^{2}$ pixel spacing. T1 images were acquired using a multiecho multiplanar spin echo sequence with the following settings: 85° flip angle, 5 mm slice thickness, repetition time of 333.3 ms, echo time of 8 ms, echo train length: 1, bandwidth of 81.4 Hz/px, and $1.17\times 1.17\;{\text{mm}}^{2}$ pixel spacing. T2 images were acquired using a fast recovery fast spin echo sequence with the following parameters: 90° flip angle, 5 mm slice thickness, repetition time of 2.37×103 ms, echo time of 109.87 ms, number of averages 3, echo train length: 27, bandwidth of 122.07 Hz/px, SAR: 1.16 W/kg, and $0.586\times 0.586\;{\text{mm}}^{2}$ pixel spacing. Two‐dimensional FIESTA cine images were acquired using a fast gradient echo readout with the following parameters: 75° flip angle, 5 mm slice thickness, repetition time of 7.1 ms, echo time of 1.96 ms, inversion time: 200 ms, bandwidth of 651.0 Hz/ px, SAR: 1.4186 W/kg, and $1.17\times 1.17\;{\text{mm}}^{2}$ pixel spacing. These values are summarized in Table 1. The total time the films were exposed to the magnetic field was 35 min.

**Table 1:** MR imaging parameters

	*Localizer*	*T1*	*T2*	*Cine*
Pixel Spacing (${\text{mm}}^{2}$)	1.5625×1.5625	1.17×1.17	0.586×0.586	1.17×1.17
Flip Angle (deg)	50	85	90	75
Slice Thickness (mm)	8	5	5	5
Repetition Time (ms)	3.233	333.3	$2.37\times 10^{3}$	7.1
Echo Time (ms)	1.352	8	109.87	1.96
Echo Train Length	N/A	1	27	N/A
Inversion Time (ms)	N/A	N/A	N/A	200
Bandwidth (Hz/px)	976.6	81.4	122.07	651
Averages	1	1	3	1

### E. Specific absorption rate (SAR)

A second experiment was performed to determine the effect of specific absorption rate (SAR) on the film. In MRI, the radiofrequency (RF) pulses used to excite the spins also deposit RF energy that may cause unwanted heating. The heating is measured by specific absorption rate,

(1) $$\text{SAR}\propto B^{2}\times \theta^{2}\times \text{BW}$$

where *B* is the magnetic field strength, θ is the flip angle, and *BW* is the RF bandwidth.^(12)^ Regulatory guidelines restrict the amount of SAR deposited in a patient to 4 W/kg to the whole body averaged over a 15 min exposure. It has been demonstrated that changes in temperature during irradiation and scanning of Gafchromic film can lead to inaccurate measurements.^(7)^

SAR experiment films were placed in the magnetic field for approximately 15, 30, and 45 min and received cumulative whole‐body and peak SAR of 2.32 and 4.65 W/kg (SAR low), 3.15 and 6.32 W/kg (SAR medium), and 5.39 and 10.81 W/kg (SAR high), respectively, through a series of applied pulse sequences. The SAR values were derived from the GE sequence‐specific SAR calculation. Films were placed on a human thoracic cavity‐sized anthropomorphic phantom, composed of foam. Approximately, 50, 35, and 20 min passed between being exposed to the magnetic field and the irradiation for the low, medium, and high exposure of SAR films. Irradiation took approximately 20 min to complete starting from the lowest dose to the highest (0, 5, 10, 25, 100, 200 cGy).

## III. RESULTS

**Figure 1:**
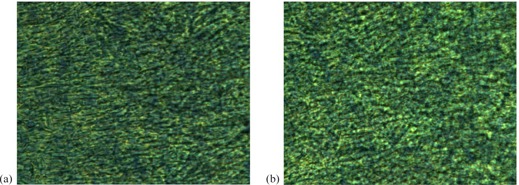
Microscopic images of irradiated Gafchromic film: (a) without exposure to the magnetic field; (b) with exposure to the magnetic field. The polymerization process undergone by the LiPCDA darkens the film, as demonstrated in both images. However, the film unexposed to the magnetic field (a) shows more clumping of the darkened polymers and an overall darker coloration, compared to the exposed film (b).

No discernable preferential alignment was detected between the microscopic images of the irradiated film unexposed and exposed to the magnetic field. Figure 1 illustrates that photo‐polymerization occurred for both films. However, the film unexposed to the magnetic field, Fig. 1(a) shows more clumping of the darkened polymers and an overall darker coloration compared to the exposed film, Fig. 1(b). However, computer vision edge detection techniques were unable to differentiate the differences between the images. These results were mirrored by the quantitative analysis of the red‐channel dosimetry described below.

**Figure 2:**
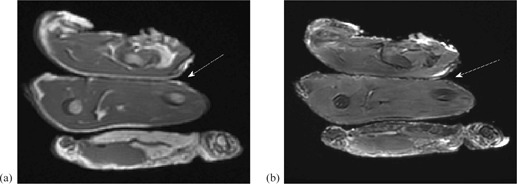
Artifact‐free MR images with Gafchromic film between the slices of chicken: (a) T1‐weighted MR image illustrating the lack of imaging artifacts associated with Gafchromic film; (b) T2‐weighted MR image depicting the lack of imaging artifacts associated with Gafchromic film. The films are located at the layer indicated by the arrows.

Qualitative MR image analysis was performed by visually observing each imaging slice acquired using the MRI imaging sequences described above. Figure 2 is a representative example of the collected images and demonstrates the lack of imaging artifacts observed on the T1‐weighted images (Fig. 2(a) and T2‐weighted images (Fig. 2(b)). No imaging artifacts due to the Gafchromic film were present in any of the images. The T1‐ and T2‐weighted images were acquired using institutional standard pulse sequences for T1‐ and T2‐weighted imaging.

**Figure 3:**
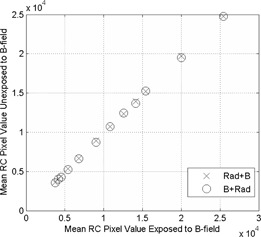
Correction curve for offset in pixel value due to MR field. The mean red‐channel pixel value from the control (unexposed to B‐field) films is plotted as a function of mean pixel value from the two experimental setups: irradiation then magnetic field (x), and magnetic field then irradiation (o). A linear regression was performed to correct for magnetic field, where y=1.02×x, where y represents the unexposed data and x the exposed data, with $r^{2}=0.9998$ and $r^{2}=0.9997$ for RAD+B and B+RAD, respectively.

The red channel reading for each film was extracted for analysis. Pixel variations across the measured area between two films that underwent the same experimental conditions did not exceed 1.5% for all measurements made. Linear regression with a forced zero intercept was performed on the control versus the RAD+B and the control versus the B+RAD. Both regressions yield ${\text{RC}}_{\text{control}}=1.02\times ({\text{RC}}_{B+\text{RAD}}\;\text{or}\;{\text{RC}}_{\text{RAD}+B})$. The $r^{2}$ values were 0.9998 and 0.9997 for control versus RAD+B and control versus B+RAD, respectively (Fig. 3). This pixel value correction method has been used in the following analysis to counteract the effect of magnetic field on the film.

Tables 2 and 3 summarize the dose difference between the control, the uncorrected, and the corrected pixel readings. The same dose calibration curve was used converting the pixel value to dose. Using the uncorrected RAD+B data led to a maximum difference and maximum percent difference of 41.0 cGy or 5.1% for 800 cGy and 1.1 cGy or 11.4% for 10 cGy. All other dose points varied from 10.9% to 6.0%. When the correction was applied, the maximum difference and maximum percent difference were reduced to 11.4 cGy or 1.4% for 800 cGy and 1.17 cGy or 23.4% for 5 cGy. All other dose point differences were less than 3%. A similar trend was observed for the B+RAD data. The maximum dose difference of 41.1 cGy or 5.1% for 800 cGy and 2.4 cGy or 24.2% for 10 cGy was reduced to 11.5 cGy or 1.4% and 1.2 cGy or 12.1% after the correction.

Using the same prior determined correction factor, the corrected dose from the SAR experiment is presented in Table 4. Since the true irradiated dose was the same for all three SAR experiments, there was a clear trend of increasing dose overestimation with increasing SAR. The correction factor derived from the first experiment, which belonged to the "low‐SAR" category, was effective only for the "low‐SAR" measurements.

**Table 2:** Uncorrected and corrected B+RAD dose measurements

*Dose (cGy)*	*Uncorrected* B+RAD *(cGy)*	*Corrected* B+RAD *(cGy)*
0.0	−2.0	−1.0
5.0	4.0	5.1
10.0	7.6	8.7
25.0	22.1	23.8
50.0	46.7	49.2
100.0	91.8	96.0
150.0	143.0	149.1
200.0	195.6	203.7
250.0	237.4	247.0
300.0	295.2	307.0
500.0	477.3	496.0
800.0	758.9	788.5

**Table 3:** Uncorrected and corrected RAD+B dose measurements

*Dose (cGy)*	*Uncorrected* RAD+B *(cGy)*	*Corrected* RAD+B *(cGy)*
0.0	−1.2	−0.0
5.0	5.0	6.2
10.0	8.9	10.1
25.0	23.5	25.3
50.0	47.7	50.3
100.0	96.3	100.6
150.0	143.6	149.7
200.0	192.0	199.9
250.0	246.3	256.2
300.0	293.0	304.8
500.0	488.3	507.5
800.0	759.0	788.6

**Table 4:** Corrected dose measurements for films exposed to different levels of SAR

*Dose (cGy)*	*Low SAR (cGy)*	*Medium SAR (cGy)*	*High SAR (cGy)*
0.0	0.4	2.8	4.7
5.0	5.3	7.2	8.7
10.0	10.7	12.5	14.8
25.0	24.8	27.0	28.6
100.0	102.4	104.9	108.5
200.0	199.5	207.5	217.3

## IV. DISCUSSION

The results have shown that it is feasible to use Gafchromic EBT2 film for absolute dosimetric measurements made with MRI‐radiotherapy systems. An underdose effect was clearly demonstrated in the films that were exposed to the magnetic field. This effect was also reported by the Utrecht group.^(8)^ In order to have accurate readings with Gafchromic film a correction must be applied to the original pixel values. After correction, the measured dose was shown to be within 1.5%/1.5 cGy from the dose recorded by the control group. The method presented for correction required acquisition of a full set of data for calibration from films both exposed and unexposed to the magnetic field. Due to the limited energy dependency of Gafchromic film, the application of this calibration/correction procedure should be feasible for both the MR/linac (6 MV) and ViewRay (cobalt‐60) systems, in conjunction with any other alternate source of radiation.

However, precautions should be taken when generating the correction curve, as the selected imaging sequences used while exposing the films to the magnetic field will affect the SAR deposited on the films. While the effect of SAR was not characterized in this paper, a clear trend was demonstrated. Increasing SAR led to an increase in red‐channel pixel value; therefore, correction factors based on the red channel readings will be SAR‐dependent. Thus, imaging sequences closest to those actually used in clinical applications should be implemented when generating correction data. When SAR is appropriately matched to the correction curve, the maximum difference and maximum percent difference measurements were minimal. It is expected that the low‐SAR technique, which reflects the application of localizers and three traditional pulse sequences (approximately 15 min of imaging) is the most like type of imaging to be adopted by MR‐based IGRT.

Vendors often change parameters within pulse sequence based on patient weight in order to meet the FDA's SAR limits, which should also be kept in mind when generating correction data. Additionally, the nature of the eddy currents that induce the heating associated with SAR are dependent on the object being imaged, so a phantom made of an appropriate material and sized similarly to a person should be selected when generating the correction curve. Using a set imaging protocol may be the best approach to generating the correction curve and accurately measuring dose. However, SAR is known to change when metal implants are in the vicinity of the induced eddy current. The effect this has on the dosimetric measurements made with Gafchromic film should be further studied.

The experiments presented herein did not make use of concurrent radiation and magnetic field exposure of the Gafchromic film. The lack of concurrent exposure means the fast polymerization process was not completely characterized by the data presented in this paper. Further studies should be performed to characterize the effect of the fast polymerization processes when exposed to a magnetic field. Additionally, this study did not utilize multichannel (red, green, blue) film dosimetry, which may have less dependence on changes in temperature than red‐channel dosimetry. A correction method for multichannel pixel values is still pending for future investigation.

## V. CONCLUSIONS

Gafchromic EBT2 film does not introduce imaging artifacts in MRI images. The effect of magnetic resonance imaging on Gafchromic EBT2 film has been partially characterized for clinical radiation therapy dosimetry use, and a correction method has been presented. However, full characterization of the effect of MRI on Gafchromic film remains for future studies. By applying a correction factor to the red‐channel pixel value prior to dose conversion, accurate dosimetry can be restored. Based on experimental results, the use of Gafchromic film for *in vivo* dosimetric applications with an MR/linac or ViewRay is clinically feasible and does not cause MR imaging artifacts.
